# Advancing the beneficial use of machine learning in health care and medicine: Toward a community understanding

**DOI:** 10.1371/journal.pmed.1002708

**Published:** 2018-11-30

**Authors:** Linda Nevin, on behalf of the PLOS Medicine Editors

**Affiliations:** Public Library of Science, San Francisco, California, United States of America and Cambridge, United Kingdom

Due to the abundance of health data and growing computational power, machine learning (ML) is engaging health researchers in a process of discovery around developing data-driven algorithms to make clinically reliable predictions. ML has the potential to provide effective tools to improve outcomes and reduce costs in health care, and the clinical community should partake in developing and evaluating these discoveries. However, if misguided applications are to be avoided, methodological savvy will be needed to develop, interpret and implement ML in medicine [1,2].

In preparing *PLOS Medicine’s* Special Issue (SI) on Machine Learning in Health and Biomedicine, Guest Editors Atul Butte, Suchi Saria, and Aziz Sheikh, and the *PLOS Medicine* Editors, have identified two principles in the design and reporting of ML studies that we believe should guide researchers in advancing the beneficial use of ML in healthcare and medicine. These principles, which also inform *PLOS Medicine’s* editorial priorities for manuscript submissions in this field, require first, that models derived through ML are demonstrably fit for their stated clinical purpose, and second, that researchers undertake and report appropriate efforts to validate these models in external datasets.

## Fit-for-purpose performance

An ML model need not be ready for off-the-shelf, practice-changing implementation to make a valuable contribution, but must achieve a clear purpose. The current SI includes reports on ML approaches that have undergone retrospective validation and are now ready for prospective testing, ML at early stages of validation, and head-to-head comparisons between standard epidemiological and ML approaches that suggest future directions without themselves establishing clinical utility. The articles vary widely in the intended application or “use cases” for their models. In a study from Soo-Jin Kang and colleagues, ML deploying imaging data from intravascular coronary angiography was used to diagnose coronary ischemia without a more invasive measurement—fractional flow reserve—that is currently the diagnostic standard [3]. The authors were not trying to challenge the standard of care, but to improve diagnosis when resource constraints or clinical indications make fractional flow reserve impractical or unsuitable. For applications intended to provide pragmatic options for nonideal circumstances, model performance can be benchmarked against current practice rather than recommended practice, as long as the limitations of the advance are clear to readers.

Early research planning should consider clinically acceptable performance characteristics for the targeted application, and a clear description of its intended use—and inappropriate potential uses—is essential. In another study in this SI, Andrew Taylor and colleagues developed a convolutional neural network (CNN) for detection of pneumothorax on chest radiography. The trained model had lower sensitivity (in the 0.8 range) than specificity (in the 0.9 range) for detection of moderate and large pneumothoraces [4], suggesting that a health system which relied on this model as a replacement for radiologist assessment would fail to diagnose an unacceptable proportion of urgent cases. However, the study was designed to develop an additive alert system to be implemented at image acquisition, particularly in settings where radiologist assessment occurs hours or even days later. With this system, the researchers aimed to detect moderate and large pneumothoraces needing immediate attention, while keeping specificity high to avoid “alert fatigue” among radiologists. This intended use case is certified by Taylor and colleagues’ date-stamped prespecified plan for the project, provided as Supporting Information with the article. In order to to minimize misinterpretation of exploratory analyses, *PLOS Medicine* requires authors to provide a prospective analysis plan, if one was used, for observational studies [5]. In ML—where exploratory comparisons are a given—researchers should accordingly develop evidence-based expectations for clinically acceptable performance, and thresholds for external validity, in advance of assessing the model’s outcomes. The development of a pre-specified ML analysis plan (as yet unseen in submissions to this journal) represents a potential standard for ML researchers who are planning research with clinical applicability.

## External validation

An ideal scenario for development and validation of prediction models, best suited to multisite studies, is one in which, first, data from the development sample are partitioned non-randomly—e.g., by site, department, geography, or time—and each subset is held out in turn to test the performance of models developed on pooled data from the remaining subsets [6]. If these models perform well, the final model can then be developed using all available data. Because the partitions are non-random, this approach is considered a type of external validation and increases confidence in model generalizability. The model can then be tested in entirely separate datasets as they become available; validation in datasets with similar characteristics provides evidence for reproducibility of the model's performance, and validation in divergent datasets—ideally differing in participant characteristics, potential biases, confounders, and practice patterns—assesses the potential for model transportability.

When adequate datasets are available, this rigorous and principled approach should yield robust prediction models with little propensity to reflect noise or bias. ML provides no exception to the need for validation—indeed, their ability to identify nonlinear associations may render ML approaches particularly susceptible to overfitting. In a further study from the SI that used ML to detect pneumonia on chest radiography, Eric Oermann and colleagues found that a CNN model trained on pooled data from two large U.S. hospital systems could not replicate its performance when tested on data from a third hospital system [7]. In further analyses, the researchers found evidence that this model exploited imperceptible (to humans) image features associated with hospital system and department, to a greater extent than image features of pneumonia, and that hospital system and department were themselves predictors of pneumonia in the pooled training dataset. Thus, when tested in an independent hospital system the model may have been deprived of predictors that were key to initial fitting but irrelevant to patient diagnosis.

This demonstration of potential confounding has heightened our attention to the rigor of validation, already an editorial priority for reports of diagnostic tests intended for clinical use. Validation, like performance, must be fit for purpose, with highest standards applying when clinical decisions are implicated. In one SI study, Yizhi Liu and colleagues used electronic medical record (EMR) data to develop and validate a Random Forest model to estimate risk of future high myopia among Chinese school-aged children [8]. The model was trained (with internal cross-validation) using data from one large ophthalmic center in China, and then externally validated in a dataset pooled from seven additional centers. The researchers further tested their model’s performance on data from two longitudinal cohort studies, to better understand generalizability across different types of datasets. Thorough and resourceful validation of this kind should be highly sought by medical journals seeking to publish conclusive advances in ML.

Studies based on EMR and registry datasets are commonly amenable to performance validation using temporally or geographically distinct patient subsets. In a single-site study using ML to estimate risks of surgical complications, Corey and colleagues, with the intent of validating a data curation tool within their own center, used the most recent 5 months’ data from their repository for validation because these data best represented up-to-date patient characteristics and medical practices at their center [9]. In another SI study, Fatemeh Rahimian and colleagues estimated emergency admissions at the population level using ML with data from the UK Clinical Practice Research Datalink, with data from two northern districts of England held out for model validation [10]. The use of geographic partitioning increases confidence that ML predictions do not rely on district-specific features. A pre-specified analysis plan that sets out the partitioning scheme can avoid the appearance of post-hoc selection in data partitioning by establishing that choices were based on the model’s intended purpose, before sensitivity and specificity from internal validation were known.

In the evaluation of research for this Special Issue, the *PLOS Medicine* Editors attained increased confidence in ML’s potential to advance care, but also identified a need for clearer standards for ML study design and reporting in medical research. We hope these published articles provide a resource that assists ML researchers in finding the shortest path to improving human health on a broad scale, and we look forward to publishing future research in this dynamic area.

## References

[1] Schulam P, Saria S. Reliable decision support using counterfactual models. In: Guyon I, Luxburg UV, Bengio S, Wallach H, Fergus R, Vishwanathan S, Garnett R, editors. Advances in Neural Information Processing Systems 30; 2017 p. 1697–1708.

[2] Subbaswamy A, Saria S. Counterfactual Normalization: Proactively Addressing Dataset Shift Using Causal Mechanisms. Uncertainty in Artificial Intelligence; 2018 p. 947–957. Available from: https://arxiv.org/abs/1808.03253

[3] Hae H, Kang S-J, Kim W-J, Choi S-Y, Lee J-G, Bae Y, et al Machine learning assessment of myocardial ischemia using angiography: Development and retrospective validation. PLoS Med. 2018;15(11):e1002693 10.1371/journal.pmed.1002693 30422987 PMC6233920

[4] Taylor AG, Mielke C, Mongan J. Automated detection of moderate and large pneumothorax on frontal chest X-rays using deep convolutional neural networks: A retrospective study. PLoS Med. 2015;15(11):e1002697 10.1371/journal.pmed.1002697 PMC6245672 30457991

[5] The *PLOS Medicine* Editors. Observational Studies: Getting Clear about Transparency. PLoS Med. 2014;11(8):e1001711 10.1371/journal.pmed.1001711 25158064 PMC4144975

[6] Steyerberg EW, Harrell FE Jr. Prediction models need appropriate internal, internal–external, and external validation. J Clin Epidemiol. 2016;69:245–7. 10.1016/j.jclinepi.2015.04.005 25981519 PMC5578404

[7] Zech JR, Badgeley MA, Liu M, Costa AB, Titano JJ, Oermann EK. Variable generalization performance of a deep learning model to detect pneumonia in chest radiographs: A cross-sectional study. PLoS Med. 2018;15(11):e1002683 10.1371/journal.pmed.1002683 30399157 PMC6219764

[8] Lin H, Long E, Ding X, Diao H, Chen Z, Liu R, et al Prediction of myopia development among Chinese school-aged children using refraction data from electronic medical records: A retrospective, multicentre machine learning study. PLoS Med. 2018;15(11):e1002674 10.1371/journal.pmed.1002674 30399150 PMC6219762

[9] Corey KM, Kashyap S, Lorenzi E, Lagoo-Deenadayalan SA, Heller K, Whalen K, et al Development and validation of machine learning models to identify high-risk surgical patients using automatically curated electronic health record data (Pythia): A retrospective, single-site study. PLoS Med. 2018;15(11):e1002701 10.1371/journal.pmed.1002701 30481172 PMC6258507

[10] Rahimian F, Salimi-Khorshidi G, Payberah AH, Tran J, Ayala Solares R, Raimondi F, et al Predicting the risk of emergency admission with machine learning: Development and validation using linked electronic health records. PLoS Med. 2018;15(11):e1002695 10.1371/journal.pmed.1002695 30458006 PMC6245681

